# Correlates of Burnout Profiles in Chinese Nurses: A Network Analysis of Psychological and Occupational Factors

**DOI:** 10.1155/jonm/6714188

**Published:** 2026-10-05

**Authors:** Dan Wang, Yuanyuan La, Wanyang Li, Bodi Zhang, Kui Fang, Jiefu Yu

**Affiliations:** ^1^ School of Government Beijing Normal University 19 Xinjiekou Wai Street Beijing 100875 China bnu.edu.cn; ^2^ Department of Social Medicine School of Public Health Shanxi Medical University Taiyuan City Shanxi 030001 China sxmu.edu.cn; ^3^ Department of Nutrition Qilu Hospital of Shandong University Jinan 250012 Shandong China qiluhospital.com; ^4^ School of Sociology Beijing Normal University 19 Xinjiekou Wai Street Beijing 100875 China bnu.edu.cn; ^5^ Department of Neurosurgery The First Affiliated Hospital of China Medical University 155 Nanjing North Street Shenyang 110001 Liaoning China cmu.edu.cn

**Keywords:** burnout, latent class analysis, network analysis, occupational health, perceived stress, resilience

## Abstract

**Objectives:**

This study aims to reveal the heterogeneous structure of job burnout among Chinese nurses, identify its key psychological and occupational influencing factors, and analyze the dynamic relationship network among various dimensions within these factors, with the expectation of providing a basis for formulating precise intervention measures.

**Methods:**

A cross‐sectional survey of 1133 registered nurses from China was conducted. Participants completed validated scales of psychological resilience, perceived stress and burnout, and self‐designed job‐related questions. Latent class analysis (LCA) is applied to categorize nurses′ manifestations of job burnout. Through multinomial regression analysis of influencing factors, key predictive factors that distinguish different burnout categories are determined. Finally, network analysis methods are applied to construct and analyze the correlation network among internal variables of key factors, identifying core nodes within the network.

**Results:**

The mean scores for the Maslach Burnout Inventory (MBI), Connor‒Davidson Resilience Scale (CD‐RISC), and the Perceived Stress Scale (PSS) were 57.27 ± 19.47, 23.27 ± 9.35, and 17.37 ± 5.89, respectively. LCA revealed a 3‐class solution: a mild burnout group (37.21%), a high emotional exhaustion group (47.49%), and a low personal accomplishment group (15.30%). Female nurses and those with higher resilience were less likely to belong to the high emotional exhaustion class, whereas unmarried status, working in internal medicine, low income, and low physical activity increased the risk. Network analysis identified PSS10 (“Difficulties piling up”), PSS1 (“Upset unexpected”), and RISC8 (“Coping with stress”) as the most central nodes in terms of strength. Strong negative connections were observed between resilience and perceived stress clusters, with RISC4 (“See myself as a strong person”) and PSS4 (“Handle personal problems”) being notably linked. Job factors showed weaker but meaningful connections to stress nodes. Bootstrap difference tests confirmed that key centrality values were significantly higher than those of peripheral nodes (*p* < 0.05), and the network demonstrated high stability (CS‐coefficient > 0.5).

**Conclusions:**

There is significant group heterogeneity in job burnout among Chinese nurses. Psychological resilience, perceived stress, and job‐related factors are core variables that lead to different patterns of burnout, and these variables have a closely intertwined dynamic relationship network within them. Some core dimensions may become key targets for intervention. The study offers a framework that progresses from classification to mechanism, informing the understanding and targeted intervention of nurse burnout.

**Implication for Nursing Management:**

Applying the job demands–resources (JD‐R) model to our network findings, we translate statistical centrality into management priority: perceived stress (a key demand) and psychological resilience (a key resource) are the most influential drivers of the burnout network. Effective nursing management should therefore pursue parallel strategies—reducing demands (e.g., workload calibration and shift predictability) while simultaneously strengthening resources (e.g., resilience workshops, peer debriefing, and supervisory support). In addition, our profile‐based results call for differentiated protocols: emotional exhaustion signals demand overload and requires immediate operational relief, whereas low personal accomplishment signals resource scarcity and requires developmental engagement (e.g., training, mentoring, and recognition). Because these dimensions interact, even focused modifications in organizational infrastructure can produce widespread improvements.

## 1. Introduction

The occupational health of medical personnel is a global public health issue, and nursing is universally recognized as one of the most demanding professions within the healthcare sector [1]. Nursing is a profession characterized by high levels of stress and emotional demand, making burnout a pervasive and serious occupational hazard [2]. Globally, up to 11.23%–30.7% of nurses report high levels of burnout [1], which not only compromises patient safety but also fuels turnover intentions and workforce shortages [3]. China faces a high prevalence of burnout among nurses—a problem that erodes care quality, endangers patient safety, and accelerates the exodus of nursing professionals [4]. In 2021, Guo et al. conducted a meta‐analysis on the detection rate of occupational burnout among nurses in China, and the results revealed that the burnout rates in the dimensions of emotional exhaustion (EE), depersonalization (DP), and reduced personal achievement (PA) were 11.34%, 7.40%, and 64.02%, respectively [5]. Chinese nurses have a high incidence rate of occupational burnout, which not only affects the quality of nursing services and patient safety but also exacerbates the loss of nursing talent.

High prevalence rates of burnout among nurses have been consistently documented globally, leading to severe individual and organizational consequences, including increased staff turnover, heightened risk of medical errors, and substantial healthcare costs [6, 7]. Effectively addressing this issue is therefore a critical priority for healthcare systems. Burnout is not a monolithic experience but a heterogeneous psychological syndrome, classically defined by Maslach’s three dimensions: EE, DP, and reduced PA [8]. These components can combine in varying patterns, forming distinct burnout profiles or subtypes, e.g., high exhaustion only, classic burnout across all dimensions, or disengaged [9]. Recognizing this heterogeneity is crucial, as different profiles likely have different etiologies and may require tailored, profile‐specific intervention strategies rather than a one‐size‐fits‐all approach.

Perceived stress and resilience were selected as key psychological variables for two main reasons. First, previous studies have consistently identified perceived stress as one of the strongest proximal correlates of burnout among nurses, with higher stress levels consistently associated with greater EE and DP [10–13]. Second, resilience has been proposed as a protective factor that may buffer the negative effects of occupational stressors on burnout [14, 15]. Meta‐analytic evidence suggests that higher resilience is associated with lower burnout scores across healthcare settings [16]. Despite these established bivariate associations, little is known about how perceived stress and resilience operate jointly with occupational factors to shape distinct burnout profiles among clinical nurses. Therefore, we included both variables to examine their unique and combined associations with burnout patterns.

Despite this understanding, prevailing research on predictors of nurse burnout has notable limitations. First, most studies rely on traditional regression–based methods, which, while identifying independent predictors, fail to capture the complex, reciprocal interactions within the system of contributing factors [17]. Second, investigations about Chinese nurses often examine psychological factors (e.g., resilience and perceived stress) and occupational factors (e.g., workload, job control, and team support) in isolation [18, 19]. This fragmented view overlooks how these domains dynamically influence each other to culminate in burnout. A more integrative, systems‐oriented analytical framework is needed to elucidate this complexity.

Network analysis offers a powerful paradigm to address these gaps [20]. As an emerging psychometric methodology, it conceptualizes psychological constructs as interconnected nodes within a system. By modeling partial correlations, it can visualize the intricate web of direct and indirect relationships among multiple variables, moving beyond prediction to mapping structure [21]. Crucially, it can identify central nodes—factors with the strongest and most numerous connections—which may act as core drivers of the system and represent high‐potential targets for intervention.

Building on this rationale, the present study aims to provide a nuanced, system‐level understanding of burnout in Chinese nurses through a sequential analytical approach. The study has two primary objectives: (1) to identify distinct burnout profiles among nurses using latent profile analysis (LPA) and (2) to construct and analyze a psychological‐occupational factor network to elucidate the interrelationships among key correlates (e.g., resilience, perceived stress, and workload) within the identified profiles.

Although burnout among nurses has been extensively studied, prior research has largely examined its risk factors in isolation, leaving the interplay among external work conditions, cognitive appraisals, and personal resources underexplored. To address this gap, we draw upon the job demands–resources (JD‐R) model [22] and Lazarus and Folkman’s transactional stress theory [23] to frame a unified conceptual model. Within this framework, job factors (e.g., workload, employment type, and work environment risk) represent external job demands; perceived stress reflects the cognitive appraisal of these demands as threatening or overwhelming; and psychological resilience serves as a key personal resource that buffers the stress–strain relationship. These three tiers are theoretically interlinked rather than independent. However, studies simultaneously modeling their network relationships remain scarce. Network analysis is particularly suited to delineate this complexity by visualizing conditional dependencies without prespecified causal pathways [24]. Accordingly, this study constructs a network model integrating job factors, perceived stress, and resilience to identify central drivers of nurse burnout and inform targeted management interventions.

## 2. Methods

### 2.1. Study Design and Setting

This was a cross‐sectional survey conducted via an anonymous, self‐administered online questionnaire. Data were collected via a structured questionnaire distributed through an online platform—Wenjuanxing (Questionnaire Star), a widely used professional online survey tool in China with functions of data encryption and real‐time data management to ensure data security and accuracy. The questionnaire was designed on the basis of validated scales in previous literature and adjusted for the nursing context and pretested for content validity and reliability with a pilot test involving 20 nurses, resulting in a Cronbach’s *α* coefficient of 0.82–0.93 for the main scales, indicating good internal consistency.

Data collection was conducted from June 2025 to August 2025. The survey was distributed through online nursing workgroups across mainland China. These workgroups consisted of nurses from various healthcare institutions, including tertiary hospitals, secondary hospitals, and primary care facilities, covering different clinical departments (e.g., internal medicine, surgery, emergency, and intensive care units). The workgroups were accessed through collaboration with local nursing associations, hospital nursing departments, and head nurses, who disseminated the questionnaire link via WeChat (a dominant social media platform in China) workgroups to eligible nurses. The participants completed the questionnaire voluntarily online, with no time or location restrictions other than internet access.

### 2.2. Patient Eligibility Criteria

Participants were eligible if they met the following inclusion criteria: (1) registered nurses with a valid nursing license, (2) currently engaged in clinical nursing work (excluding administrative or retired nurses), (3) able to understand and complete the online questionnaire independently (without cognitive or language barriers), and (4) voluntarily agreed to participate in the study.

The exclusion criteria were as follows: (1) nursing students or intern nurses without formal employment, (2) nurses on long‐term leave (≥ 3 months) during the survey period, and (3) incomplete questionnaire responses (defined as missing > 10% of key items, on the basis of predefined criteria).

### 2.3. Sampling and Recruitment

A convenience sampling method was used to recruit 1173 participants; 40 were excluded for failing to complete at least 90% of the survey items, yielding a final analytical sample of 1133. The questionnaire link, along with a brief introduction of the study (purpose, confidentiality, and estimated completion time), was distributed through WeChat workgroups of nurses. These workgroups were identified through professional networks, local nursing associations, and hospital nursing management teams to ensure coverage of diverse nursing settings (e.g., urban vs. rural hospitals; general vs. specialized hospitals). The participants were informed of the study’s voluntary nature, anonymity (no personal identifiers were collected, such as name or employee ID), and the right to withdraw at any time without consequences.

### 2.4. Ethical Considerations

Ethical approval was obtained from the Medical Science Research Ethics Committee of the First Affiliated Hospital of China Medical University (Approval No. 2025–595), and all participants provided informed consent by checking a box before starting the questionnaire in accordance with the Declaration of Helsinki.

### 2.5. Measures

#### 2.5.1. Maslach Burnout Inventory (MBI)

MBI was used to assess occupational burnout, developed by Maslach et al. [25] and revised by Zhang et al. [26]. The MBI comprises 22 items rated on a 7‐point Likert scale from 0 (*never*) to 6 (*every day*). It measures three dimensions of burnout: EE (9 items, 0–54 points), DP (5 items, 0–30 points), and PA (8 items, 0–48 points). MBI operationalizes burnout as a multidimensional construct comprising three dimensions: EE (a feeling of being emotionally drained by work), DP (an impersonal, cynical attitude toward patients or the workplace), and reduced PA (a sense of low professional efficacy and achievement). These three dimensions are widely considered the core components of occupational burnout in healthcare settings. Higher scores indicate greater severity of burnout. Occupational burnout was assessed using the MBI–Human Services Survey (MBI‐HSS). Official permission to use the MBI was obtained from Mind Garden, Inc (https://www.mindgarden.com/). The Chinese version of the MBI‐HSS [27], which has been validated in previous studies[28], was used in this study. In this study, burnout was treated as a continuous variable. In the present study, the Cronbach’s *α* coefficients were 0.92 for EE, 0.83 for DP, and 0.89 for PA.

#### 2.5.2. Perceived Stress Scale (PSS)

The PSS was first developed by Cohen et al. [29] as 14 items [29]. The PSS is a self‐report instrument designed to assess perceived psychological stress over the preceding 30 days. The respondents rated each item on a five‐point Likert‐type scale ranging from 0 (*never*) to 4 (*very often*). The item scores are summed to produce a total score between 0 and 40, with higher scores indicating greater perceived stress. The PSS has good reliability and validity among Chinese nurses [30, 31]. In this study, perceived stress was treated as a continuous variable. The scale demonstrated good reliability and validity in this study (Cronbach’s *α* = 0.83).

#### 2.5.3. Connor‒Davidson Resilience Scale (CD‐RISC)

Resilience was evaluated via the revised Chinese version of the 10‐item CD‐RISC [32]. The scale contains 10 items rated on a 5‐point Likert scale (0–4, from “never” to “always”). Total scores are obtained by summing item responses, with higher scores indicating greater resilience. This scale is widely used in China and has good reliability and validity [33, 34]. In this study, resilience was treated as a continuous variable and Cronbach’s *α* = 0.96.

#### 2.5.4. Self‐Developed Occupational Exposure Index (OEI)

To comprehensively assess nurses′ level of occupational risk exposure, a composite self‐designed Nursing OEI was developed based on the JD‐R model and cumulative environmental risk framework [35]. This self‐developed composite index comprehensively assesses nurses′ level of occupational risk exposure. It comprises four core dimensions: (1) workload and time pressure, including night shift frequency, attendance rate, number of stressors, and commuting time; (2) job stability and rewards, covering employment type, income level, and professional title; (3) work environment risk, primarily based on hospital type and department category; and (4) work experience and tenure, including years of service, weekly working hours, and administrative roles. Each dimension is scored from 0 to 2 points according to the level of risk, with the total score ranging from 0 to 24 (sum of all dimension scores). Higher total scores indicate greater occupational exposure risk. Based on the total score, risk levels are categorized as follows: low risk (0–8 points), moderate risk (9–16 points), and high risk (17–24 points).

### 2.6. Statistical Analysis

Descriptive statistical analyses were conducted using SPSS version 26.0. Latent class analysis (LCA) was performed using Mplus 7.4. Network analysis was performed using *R* Version 4.3.3.

#### 2.6.1. LCA

LCA was employed to identify unobserved subgroups (latent classes) of participants based on their distinct patterns of occupational burnout. The analysis was conducted using measurements from the occupational burnout scale. A series of models ranging from one to five latent classes were fitted to the data to determine the optimal number of classes.

Model selection was guided by a combination of statistical fit indices, classification accuracy, and parsimony, as well as the theoretical interpretability of the classes [36]. The following indices were used to evaluate model fit: the Akaike information criterion (AIC), Bayesian information criterion (BIC), and sample‐size adjusted BIC (aBIC). Lower values on these information criteria indicate a better model fit [37]. The statistical significance of improvement between models with k and k‐1classes was assessed using the Lo–Mendell–Rubin likelihood ratio test (LMRT) and the bootstrapped likelihood ratio test (BLRT); a significant *p*‐value < 0.05 suggests that the k‐class model provides a better fit than the (k‐1)‐class model [36].

Classification accuracy was evaluated using entropy, with values ranging from 0 to 1. Higher entropy values (closer to 1) indicate better separation between classes, with values above 0.80 generally considered demonstrative of good classification quality [38]. In addition, the size of the smallest class was considered, as classes containing less than 5%–10% of the total sample may lack clinical or practical meaningfulness and can lead to instability in subsequent analyses [39].

#### 2.6.2. Network Estimation

The network estimation and visualization utilized the *qgraph, bootnet, and glasso* packages [24].

The inclusion of job factors (Job1–Job4), perceived stress (PSS1–PSS10), and resilience (RISC1–RISC10) as specific nodes is theoretically grounded in the JD‐R model and cognitive stress theories.

By treating these items as interconnected nodes in a system rather than aggregated sum scores, this network approach avoids masking item‐level heterogeneities and helps identify highly specific, actionable targets for clinical intervention. Edges represent regularized partial correlations between pairs of nodes, controlling for all other variables in the network. The network structure was estimated using a Gaussian graphical model (GGM) [24]. To eliminate spurious edges and ensure a sparse, interpretable network, the graphical LASSO (GLASSO) algorithm was applied in combination with the extended BIC (EBIC). The tuning hyperparameter (*γ*) was set to 0.5 to balance sensitivity and specificity in edge selection [24, 40]. In the visualization, blue solid lines indicate positive correlations, while red solid lines indicate negative correlations. The thickness and saturation of the edges indicate the strength of the associations.

To identify the core symptoms or factors within the network, four centrality indices were computed: strength (sum of absolute edge weights), closeness (inverse of the sum of the shortest paths to all other nodes), betweenness (number of shortest paths passing through a node), and expected influence (EI) (sum of raw edge weights). Higher values on these indices indicate greater importance and influence within the network.

The robustness of the network was evaluated using the bootnet package. First, nonparametric bootstrapping (2000 iterations) was used to estimate the 95% confidence intervals (CIs) for edge weights to assess accuracy. Second, the stability of centrality rankings was tested via a case‐dropping bootstrap procedure. The stability was quantified using the correlation stability (CS) coefficient, where a value above 0.5 indicates a stable and interpretable network [41]. Finally, bootstrapped difference tests were performed to determine if edges and centrality indices differed significantly from one another.

## 3. Result

### 3.1. Study Sample

A total of 1133 nurses were included in the study. The majority of nurses were female, with a mean age of 34 years; most participants held an undergraduate degree, were married, were employed on a contractual basis, reported an annual income below RMB 80000, and worked 36–45 h per week (Table 1).

**TABLE 1:** Demographic characteristics of the participants (*N* = 1133).

Variable	Values
Age, mean (SD)	34.15 (7.01)
Gender, *n* (%)	
Male	79 (6.97)
Female	1054 (93.03)
Education, *n* (%)	
Secondary vocational school	22 (1.94)
Junior college	132 (11.65)
Undergraduate	917 (80.94)
Master or above	62 (5.47)
Marital status, *n* (%)	
Married	725 (63.99)
Other	408 (36.01)
Employment relationship, *n* (%)	
Permanent staff	395 (34.86)
Contractual staff	512 (45.19)
Other	226 (19.95)
Department, *n* (%)	
ICU/emergency	140 (12.36)
Other	993 (87.64)
Title, *n* (%)	
Nurse or assistant nurse	498 (43.95)
Nurse‐in‐charge	485 (42.81)
Associate chief nurse or above	150 (13.24)
Average yearly income (RMB), *n* (%)	
< 80000	530 (46.78)
80000–120000	426 (37.60)
120000–150000	84 (7.41)
> 150000	93 (8.21)
Average weekly working hours, *n* (%)	
≤ 35	77 (6.80)
36–45	766 (67.61)
46–55	207 (18.27)
≥ 55	83 (7.33)
Perceived stress, *n* (%)	
Low	426 (37.60)
Moderate	437 (38.57)
High	270 (23.83)
Burnout, *n* (%)	
Low	381 (33.63)
Moderate	392 (34.60)
High	360 (31.77)
Resilience, *n* (%)	
Low	474 (41.84)
Moderate	308 (27.18)
High	351 (30.98)

### 3.2. Latent Class Classification

Five latent class models were fitted in this study, with their respective fit indices summarized in Table 2. Although the 4‐class model yielded the highest entropy value, one of its classes accounted for only 5.54% of the total sample, failing to meet the recommended threshold of 10% for latent class classification. Taking all fit information into account, the 3‐class model was selected as the most robust and parsimonious solution.

**TABLE 2:** Model fit indices for latent class models from one to five classes (*N* = 1133).

Class	AIC	BIC	ABIC	LMRT	BLRT	Entropy	Potential class probability
1‐class	91499.034	91720.470	91580.713	—	—	—	
2‐class	85146.067	85483.252	85270.441	0.00	0.00	0.93	55.72/44.28
3‐class	**82111.947**	**82564.883**	**82279.017**	**0.01**	**0.00**	**0.95**	**37.21/47.49/15.30**
4‐class	78709.157	79277.844	78918.923	0.02	0.00	0.961	30.07/5.54/10.12/54.26
5‐class	77085.244	77769.681	77337.706	0.08	0.00	0.94	20.21/38.84/5.38/32.66/2.91

*Note:* LMRT, Lo–Mendell–Rubin likelihood ratio test. Bold values represent the best model ultimately selected by the researcher.

Abbreviations: AIC, Akaike information criterion; BIC, Bayesian information criterion; BLRT, bootstrapped likelihood ratio test.

The probability distributions for the three classes are illustrated in Figure 1. Class 1 exhibited lower scores across all occupational burnout indicators and was thus designated as the “Mild occupational burnout group.” Individuals in Class 2 showed higher scores in the EE dimension, combined with moderate scores in DP and PA. This profile indicates a signature pattern driven primarily by severe emotional fatigue; consequently, this class was named as the “High EE group.” Class 3 participants exhibited distinctly high scores in the PA dimension (reflecting low PA), while maintaining relatively low to moderate scores in EE and DP. Therefore, this group was termed as the “Low PA group.”

**FIGURE 1:**
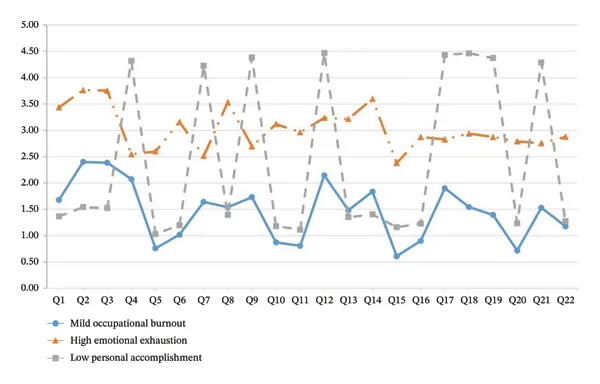
Probabilities of occupational burnout across latent classes.

### 3.3. Effects of Covariates on Odds of Nurse Burnout by Latent Class

In terms of demographic characteristics, compared to male nurses, female nurses had a significantly lower risk of belonging to the “High EE group,” whereas unmarried nurses faced a higher risk. Regarding occupational factors, nurses employed in public general hospitals and private hospitals exhibited a significantly lower risk of belonging to this group compared to the reference group. Conversely, working in the internal medicine department was associated with a significantly higher risk compared to other departments. Furthermore, relative to nurses in the highest income bracket, those in the lowest income bracket had a significantly higher risk of falling into the “High EE group.” With respect to lifestyle‐related factors, nurses with low physical activity levels had a significantly higher risk of belonging to the “High EE group” compared to those with moderate physical activity levels. In addition, longer sleep duration was associated with a lower risk of inclusion in this group. Finally, regarding psychological factors, compared to nurses reporting moderate perceived stress, those perceiving their stress as low showed a substantially reduced risk of belonging to the “High EE group.” Moreover, higher levels of psychological resilience and mindfulness were significantly associated with a decreased risk of being in the “High EE group” (Table 3).

**TABLE 3:** Multinomial regression analysis of different classes with Class 1 as reference (*N* = 1133).

Classification	Variable	Category	High emotional exhaustion group (C2)		Low personal accomplishment group (C3)	
			OR	95% CI	OR	95% CI
Demographic characteristics	Age		1.02	0.94–1.10	0.97	0.87–1.07
	Sex	Female	0.39^∗^	0.18–0.82	0.41	0.15–1.12
	Ethic	Han ethnicity	1.25	0.79–1.98	1.25	0.69–2.27
	Education	Secondary specialized school	1.09	0.26–4.65	1.67	0.17–16.81
		Junior college	0.83	0.31–2.20	2.02	0.34–11.96
		Bachelor’s degree	0.65	0.28–1.48	1.44	0.27–7.58
	Marital status	Unmarried	2.59^∗^	1.03–6.55	1.44	0.44–4.71
		Married	1.56	0.68–3.57	1.43	0.50–4.08

Occupational factors	Hospital type	Public general hospital	0.06^∗^	0.00–0.93	0.07	0.00–1.75
		Public private hospital	0.07	0.01–1.15	0.08	0.00–2.04
		Specialized hospital	0.02^∗^	0.00–0.40	0.03^∗^	0.00–0.95
		Community healthcare center	0.09	0.00–1.91	0.04	0.00–0.12
	Hospital level	Secondary hospital and below	1.00	0.61–1.66	0.86	0.46–1.63
		Grade C tertiary hospital	0.72	0.26–2.00	0.78	0.24–2.54
		Grade B tertiary hospital	1.05	0.64–1.58	0.72	0.40–1.30
	Department	Internal medicine	1.96^∗^	1.30–2.94	2.13^∗^	1.27–3.57
		Surgery	1.03	0.64–1.65	0.95	0.49–1.86
	Employment type	Permanent staff	1.34	0.21–8.78	0.96	0.86–4.86
		Contract staff	1.18	0.18–7.65	1.21	0.62–5.58
		Dispatched staff	0.82	0.12–5.52	1.02	0.75–2.21
	Professional title	Junior	0.68	0.17–2.68	1.23	0.17–9.04
		Intermediate	0.77	0.21–2.81	1.15	0.21–7.61
		Associate senior	0.80	0.32–2.97	1.09	0.33–7.21
	Years of work experience (mean ± SD)		0.97	0.90–1.04	1.03	0.94–1.13
	Average yearly income (RMB)	< 80000	2.70^∗^	1.03–7.09	4.42	0.84–23.31
		80000–120000	2.28	0.88–5.88	4.13	0.79–21.50
		120000–150000	1.82	0.64–5.12	1.68	0.27–10.65
		> 150000	2.96	0.94–9.32	1.09	0.12–10.13
	Average weekly working hours	≤ 35	1.34	0.53–3.39	2.38	0.81–7.03
		36–40	0.80	0.41–1.58	0.89	0.38–2.08
		41–45	1.22	0.61–2.42	0.83	0.35–1.98
		46–50	1.69	0.78–3.68	1.06	0.40–2.84
		51–55	2.44	0.87–6.84	1.01	0.25–4.11

Lifestyle‐related factors	Physical activity level	Low	1.64^∗^	1.23–2.21	1.48	0.98–2.25
		High	1.38	0.84–2.02	1.55	0.92–2.62
	Sleep duration (mean ± SD)		0.90^∗^	0.84–0.98	0.98^∗^	0.88–1.09
	Daily commuting time (mean ± SD)		1.00	0.99–1.01	1.01	1.00–1.01

Psychological factors	Perceived stress	Low	0.09^∗^	0.05–0.15	0.38^∗^	0.19–0.75
		High	2.18	0.60–7.91	0.25	0.04–1.70
	Resilience (mean ± SD)		0.96^∗^	0.94–0.99	0.88^∗^	0.85–0.90
	Mindfulness (mean ± SD)		0.94^∗^	0.93–0.96	0.91	0.94–1.07

^∗^ *p* < 0.05.

Regarding the “Low PA group,” the identifying occupational factors were hospital type and department. Compared to those in other types of hospitals, nurses employed in private hospitals had a significantly lower risk of belonging to this group. Furthermore, compared to their counterparts in other departments, nurses working in the internal medicine department faced a significantly higher risk of low PA. In terms of lifestyle‐related factors, a longer sleep duration was associated with a decreased risk of being categorized into the “Low PA group.” Concerning psychological factors, compared to nurses who perceived their stress as “moderate,” those with “low” perceived stress showed a substantially reduced risk of belonging to this group. In addition, higher levels of psychological resilience were found to significantly decrease the risk of being in the “Low PA group” (Table 3).

### 3.4. Network Structure

The estimated network structure of job factors, perceived stress, and resilience is presented in Figure 2. The network is fully connected, with several strong positive clusters within each dimension. Within the perceived stress cluster, the strongest associations were observed between PSS1 (“Upset unexpected”) and PSS3 (“Nervous and stressed”), and PSS1 and PSS2 (“Unable to control”). Within the resilience cluster, RISC9 (“Achieve goals”) and RISC10 (“Deal with whatever comes”) showed a robust connection.

**FIGURE 2:**
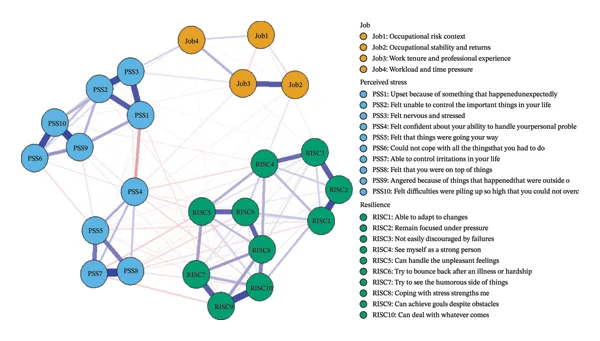
Network structure of job factors, perceived stress, and resilience.

Significant negative correlations (red edges) were found between the resilience and perceived stress clusters, most notably between RISC4 (“See myself as a strong person”) and PSS4 (“Handle personal problems”). Connections between job factors and other clusters were also identified, such as the edge between Job4 (“Workload and Time Pressure”) and the perceived stress nodes.

The centrality indices for all nodes are illustrated in Figure 3. Across the network, PSS10 (“Difficulties piling up”), PSS1, and RISC8 (“Coping with stress”) emerged as the most central nodes in terms of strength, suggesting they are the primary drivers of the network. PSS4 showed the highest value for betweenness, acting as a critical bridge between different clusters. Notably, the EI index highlighted RISC8 and PSS10 as high‐impact nodes.

**FIGURE 3:**
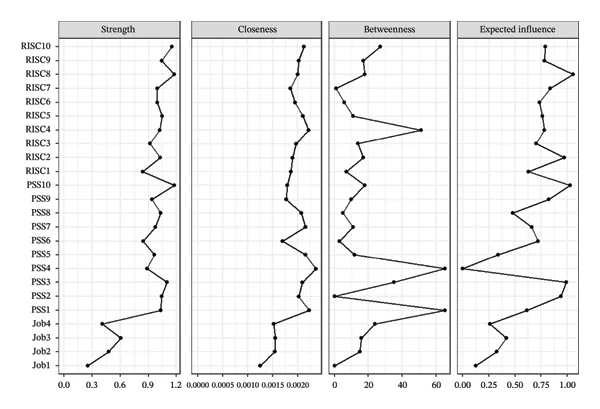
Centrality indices of job factors, perceived stress, and resilience network.

The difference tests for node strength (see Supporting Information, Figure S1) and closeness (Figure S2) confirmed that the centrality values of these key nodes were significantly higher than those of the majority of other peripheral nodes, statistically supporting their role as core components of the network.

The network demonstrated high accuracy and stability. The nonparametric bootstrap results showed relatively narrow 95% CIs for the edge weights, indicating that the estimated edge strengths are reliable (Figure 4).

**FIGURE 4:**
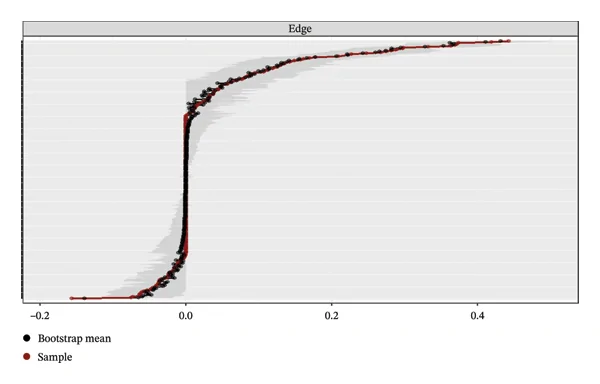
Nonparametric bootstrap confidence intervals for edge weights.

2The case‐dropping bootstrap procedure (presented in Figure 5 for centrality and Figure 6 for edges) revealed that the stability of the network was excellent. The CS‐coefficients for node strength and closeness remained consistently high even after dropping a large proportion of the sample, well exceeding the recommended threshold of 0.5. This confirms that the identified network structure and core symptoms are robust and generalizable.

**FIGURE 5:**
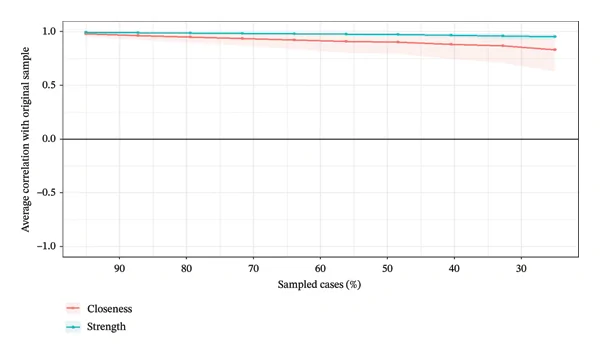
Stability of strength and closeness indices under case‐dropping bootstrap procedures.

**FIGURE 6:**
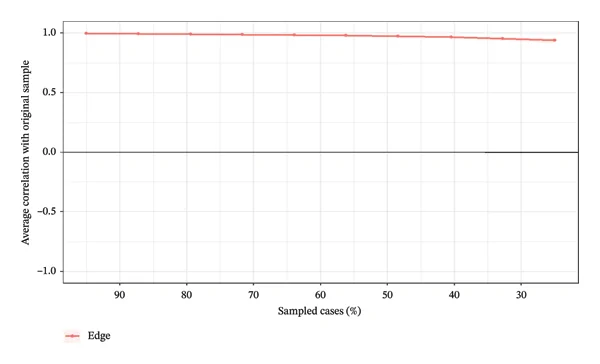
Stability of edge indices under case‐dropping bootstrap procedures.

## 4. Discussion

### 4.1. Principal Findings and Overview

This study employed an integrated person‐centered and network‐analytic approach to investigate the heterogeneity and systemic underpinnings of burnout among Chinese nurses. Three distinct burnout profiles were identified via latent profile analysis (LPA): a low burnout class (Class 1, 37.21%), a high EE class (Class 2, 47.49%), and a low PA class (Class 3, 15.30%). Multinomial regression revealed that demographic, occupational, lifestyle, and psychological factors differentially predicted membership in these profiles. Subsequently, network analysis constructed a psychological‐occupational system, identifying work experience and tenure (Work 3), specific facets of resilience (RISC9 and RISC10), and perceived stress (PSS9 and PSS10) as the most central and influential nodes within the interconnected web of factors.

### 4.2. Interpretation of Burnout Profiles and Their Correlates

The identification of a three‐class solution aligns with the established view of burnout as a heterogeneous syndrome. The largest profile, Class 2 high EE, underscores emotional depletion as the most prevalent and acute manifestation of distress in this nursing cohort. The significant correlates for this class—including working in internal medicine departments, lower income (< ¥80,000/year), low physical activity, shorter sleep duration, and higher perceived stress—paint a coherent picture of a profile driven by high job demands coupled with inadequate resources and poor recovery [42–44]. This aligns with the JD‐R model [45], where chronic demands deplete energy without sufficient compensatory rewards or recovery opportunities [45].

Conversely, Class 3 low PA represents a more cognitively‐focused profile characterized by inefficacy and reduced professional fulfillment [46]. Notably, its correlates were fewer but specific: working in internal medicine and private hospitals, shorter sleep, and lower resilience. The strong protective effect of working in private hospitals (odds ratio = 0.03) warrants further investigation but may relate to differing work cultures, patient populations, or performance metrics that influence perceptions of achievement. The central role of low resilience as a key predictor for this class highlights that feelings of ineffectiveness may be more closely tied to internal psychological resources than to external demands alone.

### 4.3. The Systemic Architecture of Burnout: Insights From Network Analysis

Moving beyond predictive associations, the network analysis provides a dynamic, systems‐level map of how key factors interact. The high centrality of work experience and tenure (Work 3) suggests it acts as a crucial hub, potentially influencing and being influenced by both stress perception and psychological resilience. This implies that interventions targeting early‐career support or modifying workload structures for seasoned nurses could have wide‐ranging ripple effects through the system.

Our centrality analyses identified perceived stress (PSS10 and PSS1) and resilience (RISC8) as the most influential nodes in the burnout network, consistent with the J‐DR model’s conceptualization of demands and resources as dual drivers of burnout. The high strength and EI of these nodes suggest that stress appraisal and coping capacity are not peripheral correlates but core engines that activate and sustain burnout across the network. Notably, PSS4 emerged as the primary bridge node (highest betweenness), indicating its unique role in connecting otherwise distinct variable clusters; this positions PSS4 as a potentially efficient intervention leverage point for disrupting maladaptive transmission pathways [47].

Importantly, the centrality of specific resilience items (e.g., RISC9 and RISC10) and stress items (e.g., PSS9 and PSS10)—rather than their global scale scores—offers a more precise and actionable intervention target. For instance, if RISC9 measures “ability to adapt to change,” then enhancing change management skills through targeted training may represent a more efficient strategy than implementing generic, nonspecific resilience programs. This item‐level precision allows nurse managers to move beyond one‐size‐fits‐all wellness initiatives toward focused, skill‐based microinterventions that directly address the most impactful nodes in the network.

Furthermore, the network structure visually demonstrates how occupational strain (work nodes) and psychological states (PSS and RISC nodes) are bidirectionally linked, breaking down the artificial dichotomy between “environmental” and “personal” factors. Rather than treating workplace conditions and individual characteristics as separate domains, our findings support a transactional model of burnout [48], wherein the individual and their work environment continuously interact and mutually shape each other over time. This has important implications for nursing management: interventions should not be siloed into “fixing the environment” or “fixing the person”; instead, they should be designed to interrupt the dynamic, self‐reinforcing loops between occupational stressors and psychological responses—for example, by simultaneously reducing workload demands while equipping nurses with tailored coping strategies to navigate the remaining challenges.

### 4.4. Comparison With Existing Literature

Our findings regarding the interplay between occupational factors, resilience, and perceived stress are broadly consistent with existing literature. Consistent with previous research [49], we found that higher perceived stress was strongly associated with greater burnout, particularly EE and DP. This aligns with studies across various healthcare settings demonstrating perceived stress as a consistent proximal correlate of burnout among nurses [50].

Regarding resilience, our results support its role as a protective factor against burnout. Nurses with higher resilience scores exhibited lower burnout levels across all three MBI dimensions. This finding is consistent with systematic review evidence showing that resilience is negatively associated with burnout and positively associated with psychological well‐being in nursing populations [51]. Meta‐analytic evidence further confirms that higher resilience is associated with lower burnout scores across diverse healthcare settings [52].

Importantly, our study extends previous bivariate findings by examining how these variables jointly operate with occupational factors to shape distinct burnout profiles. While prior research has established individual associations—perceived stress increasing burnout risk and resilience buffering against it—few studies have simultaneously modeled their combined effects using person‐centered approaches such as LCA. Our identification of heterogeneous burnout profiles suggests that the stress–resilience interplay may differ across nurse subgroups, a nuance not captured in variable‐centered analyses.

The mediating role of burnout in the stress–resilience relationship, suggested by our network analysis, is also supported by recent empirical evidence. A study among Chinese nurses found that occupational burnout mediated the relationship between perceived stress and self‐resilience, with a mediation effect accounting for 30.61% of the total effect [14]. This corroborates our finding that perceived stress and resilience are not independent predictors but rather operate through burnout as an intermediate mechanism.

### 4.5. Clinical and Organizational Implications

Our findings advocate for a precision public health approach to nurse burnout: (1) For high EE (Class 2): interventions should prioritize structural and organizational changes. This includes reviewing workload and compensation in high‐risk departments (e.g., internal medicine), implementing protected rest periods, and promoting sleep hygiene and physical activity programs.

(2) For low PA (Class 3): interventions should focus on enhancing professional efficacy and psychological resources. This could involve mentorship programs, competency‐based feedback, and targeted resilience training focusing on core components such as adaptive coping and maintaining perspective under stress (as indicated by central RISC items).

(3) Leveraging network hubs: the central nodes identified (Work 3, RISC9/10, and PSS9/10) represent high‐leverage targets. Systemically modifying these hubs (e.g., through career ladder development and cognitive‐behavioral techniques for specific stress appraisals) may be more effective than addressing peripheral factors.

### 4.6. Limitations

Several limitations must be acknowledged. First, the cross‐sectional design precludes causal inference from the network; the direction of edges is based on theory, not temporal sequence. Second, the sample, while sizable, was from a specific region of China, which may limit generalizability. The study employed a convenience sampling method through WeChat groups, which may introduce selection bias. Nurses who voluntarily responded to the online survey may differ systematically from those who did not participate. For instance, WeChat‐based recruitment likely oversampled younger, technologically adept nurses who are more active on social media platforms, while potentially underrepresenting older nurses, those in remote areas with limited internet access, or those with heavier workloads who had less time to complete the survey. Consequently, the generalizability of our findings to the entire nursing population—particularly to nurses in rural hospitals, those without smartphone access, or those in non‐Chinese healthcare systems—may be limited. Future studies using stratified random sampling or probability‐based sampling methods are needed to confirm the robustness of our findings. Third, the use of self‐report measures may introduce common method bias. Fourth, the stability of some network edges, as indicated by bootstrap confidence intervals, was suboptimal, suggesting the need for replication in larger samples. Sixth, the OEI used in this study was developed specifically for the present research and has not undergone formal psychometric validation. Although the index was designed to capture relevant occupational factors based on a review of the literature, the absence of validation evidence raises concerns about the reliability and interpretability of findings involving this variable. Therefore, results related to occupational exposure should be interpreted cautiously and as exploratory. Finally, the network did not include all possible relevant variables (e.g., specific leadership styles and social support outside work).

### 4.7. Conclusion and Future Directions

This study demonstrates that nurse burnout manifests in distinct profiles with unique predictor patterns. More importantly, by applying network analysis, it reveals the complex, interactive architecture of psychological and occupational factors that constitute these burnout states. The central nodes identified serve as empirically derived targets for intervention.

Future research should employ longitudinal network analysis to establish temporal precedence and causal pathways. Intervention studies can then be designed to “perturb” these network hubs (e.g., a trial targeting the central resilience components) and observe how changes propagate through the system, moving from correlation to causal mechanistic understanding. Such a paradigm shift from variable‐centered risk factors to system‐centered dynamic models is essential for developing effective, personalized strategies to sustain the nursing workforce.

**Nomenclature**
PSS: Perceived Stress Scale
LCA: Latent class analysis
NA: Network analysis
CD‐RISC: Connor–Davidson Resilience Scale
MBI: Maslach Burnout Inventory
SD: Standard deviation
OR: Odds ratio
JD‐R: Job demands–resources
GGM: Gaussian graphical model

## Author Contributions

Dan Wang searched the literature, conceived the study idea, designed the research framework, and wrote the original draft. Yuanyuan La performed statistical analysis, handled the questionnaire, and reported the results. Wanyang Li extracted the data and refined and modified the language. Bodi Zhang formatted and proofread the data and critically revised the study. Kui Fang verified the accuracy of the original data and revised the manuscript critically. Jiefu Yu wrote, edited, conceptualized, supervised, and administered the project.

## Funding

No funding was received for this study.

## Disclosure

All authors read and approved the final manuscript.

## Ethics Statement

Ethical approval was obtained from the Medical Science Research Ethics Committee of the First Affiliated Hospital of China Medical University (Approval No. 2025–595). Written informed consent was obtained from all participants and/or their legal guardians prior to data collection.

## Consent

Please see the Ethics Statement. All data presented in this manuscript are anonymized and no individual details, images, or videos are included. All figures and images were created originally for this manuscript. No third‐party copyrighted material has been used.

## Conflicts of Interest

The authors declare no conflicts of interest.

## Supporting Information

Additional supporting information can be found online in the Supporting Information section.

## Supporting information


**Supporting Information** Figure S1 is a bootstrap difference integration test diagram for node strength, which evaluates the importance and stability of network nodes in network analysis. RISC10, PSS10, and RISC8 have high strengths (> 1.10), indicating they may be core nodes in the network. Job1–Job4 have low strengths (< 0.61), indicating they are at the edge of the network. This diagram is used for psychological measurement network analysis, evaluating the relational network between psychological resilience (RISC), stress perception (PSS), and job‐related variables (Job). Figure S1: Bootstrapped difference integration test for node strength. The phenomenon observed in Figure S2 (Closeness) is that the overall values are very close: the closeness values of all nodes range from 0.0013 to 0.0024. Nodes with high closeness (high information propagation efficiency): PSS6 (0.0024), PSS5 (0.0021), RISC4 (0.0022), and PSS8 (0.0022). These nodes may be located at the topological center of the network. Even though their direct connections (strength) may not necessarily be the strongest, their position in the network enables them to efficiently reach other nodes. Nodes with lower closeness (relatively isolated): Job1 (0.0013), PSS1 (0.0015), Job2 (0.0015), and PSS4 (0.0016). These nodes may be at the edge of the network and require more steps to reach other nodes. Upon comparison with the strength graph, it is found that PSS10 is the most central node in the strength graph (1.20), but its closeness (0.0018) is not prominent. This indicates that it is a strongly connected local hub, but not the most efficient in global information dissemination. PSS6 is not prominent in the strength graph (0.85), but has the highest closeness (0.0024). This suggests that it may connect some key intermediary paths and is a topological hub of the network. The Job group is in a low position in both graphs, indicating that they are not only weak in direct connections but also marginal in the global network structure. Figure S2: Bootstrapped difference integration test for node closeness.

## Data Availability

The individual‐level participant data collected for this study are protected and are not available due to data privacy laws. The data are not publicly available but are held by the corresponding authors (fangkui158@126.com) and can be provided upon reasonable request and justified purpose. The dataset is fully anonymous.
