# Supplementary material for: Correlates of Burnout Profiles in Chinese Nurses: A Network Analysis of Psychological and Occupational Factors
Source: J Nurs Manag. 2026 Oct 5;2026:6714188. doi: 10.1155/jonm/6714188 (PMC13635974; doi:10.1155/jonm/6714188)
Supplement: Supplementary file 1 [file JONM-2026-6714188-s001.docx]

**Supplementary Materials**

Figure S1 is a Bootstrap difference integration test diagram for Node Strength, which evaluates the importance and stability of network nodes in network analysis. RISC10, PSS10, and RISC8 have high strengths (>1.10), indicating they may be core nodes in the network. Job1–Job4 have low strengths (<0.61), indicating they are at the edge of the network. This diagram is used for psychological measurement network analysis, evaluating the relational network between psychological resilience (RISC), stress perception (PSS), and job-related variables (Job).

**Figure S1**. Bootstrapped difference integration test for node strength

The phenomenon observed from Figure S2 (Closeness) is that the overall values are very close: the closeness values of all nodes range from 0.0013 to 0.0024. Nodes with high closeness (high information propagation efficiency): PSS6 (0.0024), PSS5 (0.0021), RISC4 (0.0022), PSS8 (0.0022). These nodes may be located at the topological center of the network. Even though their direct connections (strength) may not necessarily be the strongest, their position in the network enables them to efficiently reach other nodes. Nodes with lower closeness (relatively isolated): Job1 (0.0013), PSS1 (0.0015), Job2 (0.0015), PSS4 (0.0016). These nodes may be at the edge of the network and require more steps to reach other nodes. Upon comparison with the strength graph, it is found that PSS10 is the most central node in the strength graph (1.20), but its closeness (0.0018) is not prominent. This indicates that it is a strongly connected local hub, but not the most efficient in global information dissemination. PSS6 is not prominent in the strength graph (0.85), but has the highest closeness (0.0024). This suggests that it may connect some key intermediary paths and is at a topological hub of the network. The Job group is in a low position in both graphs, indicating that they are not only weak in direct connections but also marginal in the global network structure.

**Figure S2.** Bootstrapped difference integration test for node closeness
